# A Superaerophobic Bimetallic Selenides Heterostructure for Efficient Industrial-Level Oxygen Evolution at Ultra-High Current Densities

**DOI:** 10.1007/s40820-020-00442-0

**Published:** 2020-05-02

**Authors:** Jiaxin Yuan, Xiaodi Cheng, Hanqing Wang, Chaojun Lei, Sameer Pardiwala, Bin Yang, Zhongjian Li, Qinghua Zhang, Lecheng Lei, Shaobin Wang, Yang Hou

**Affiliations:** 1 Key Laboratory of Biomass Chemical Engineering of Ministry of Education, College of Chemical and Biological Engineering, Zhejiang University, Hangzhou, 310027 People’s Republic of China; 2 Institute of Zhejiang University - Quzhou, Quzhou, 324000 People’s Republic of China; 3 Ningbo Research Institute, Zhejiang University, Ningbo, 315100 People’s Republic of China; 4 Zhejiang Provincial Key Laboratory of Advanced Chemical Engineering Manufacture Technology, College of Chemical and Biological Engineering, Zhejiang University, Hangzhou, 310027 People’s Republic of China; 5 School of Chemical Engineering and Advanced Materials, The University of Adelaide, Adelaide, SA 5005 Australia

**Keywords:** Superaerophobicity, Bimetallic selenide, Heterostructure electrocatalyst, Strong interfacial coupling, Oxygen evolution reaction

## Abstract

Cost-effective and stable electrocatalysts with ultra-high current densities for electrochemical oxygen evolution reaction (OER) are critical to the energy crisis and environmental pollution. Herein, we report a superaerophobic three dimensional (3D) heterostructured nanowrinkles of bimetallic selenides consisting of crystalline NiSe_2_ and NiFe_2_Se_4_ grown on NiFe alloy (NiSe_2_/NiFe_2_Se_4_@NiFe) prepared by a thermal selenization procedure. In this unique 3D heterostructure, numerous nanowrinkles of NiSe_2_/NiFe_2_Se_4_ hybrid with a thickness of ~ 100 nm are grown on NiFe alloy in a uniform manner. Profiting by the large active surface area and high electronic conductivity, the superaerophobic NiSe_2_/NiFe_2_Se_4_@NiFe heterostructure exhibits excellent electrocatalytic activity and durability towards OER in alkaline media, outputting the low potentials of 1.53 and 1.54 V to achieve ultra-high current densities of 500 and 1000 mA cm^−2^, respectively, which is among the most active Ni/Fe-based selenides, and even superior to the benchmark Ir/C catalyst. The in-situ derived FeOOH and NiOOH species from NiSe_2_/NiFe_2_Se_4_@NiFe are deemed to be efficient active sites for OER.

**Electronic supplementary material:**

The online version of this article (10.1007/s40820-020-00442-0) contains supplementary material, which is available to authorized users.

## Highlights

- A superaerophobic heterostructured nanowrinkles of bimetallic selenides is developed.
- The 3D heterostructure exhibits excellent activity and stability towards oxygen evolution reaction (OER) in base.

**Electronic supplementary material:**

The online version of this article (10.1007/s40820-020-00442-0) contains supplementary material, which is available to authorized users.

## Graphical abstract

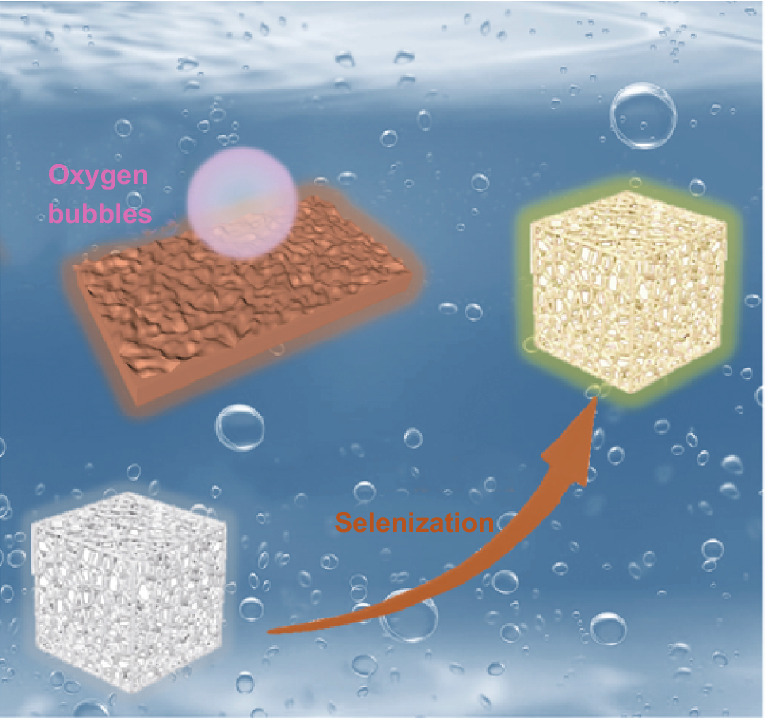

## Introduction

With the ever-worsening energy and environmental crises, electrocatalytic water-splitting is believed as a promising method to resolve the global tremendous energy needs of future societies [1, 2]. However, the sluggish reaction kinetics of four-proton-coupled electron transfer processes of oxygen evolution reaction (OER) is the bottleneck in the water-splitting process [3, 4]. Normally, the best known electrocatalysts are Ir/Ru-based materials for OER catalysis [5]. However, high cost and limited reserves largely prevent their large scale applications. Hence, development of low-cost and highly efficient non-noble metal OER electrocatalysts has been an active research in recent years, such as transition metal (Ni, Fe, or Co, etc.) phosphides [6–9], sulfides [10–16], selenides [17–20], carbides [21, 22], nitrides [23–25], hydroxides [26], oxides [27], and chalcogenides [28]. Among these reported transition metal compounds, transition metal-based selenides are specifically attractive for OER, due to their metallic nature with high intrinsic electronic conductivity. Compared with single metal-based counterparts, bimetallic selenides have been the recent focus in OER electrocatalysts, thanks to their synergistic electronic effects [29, 30].

Besides, the synergistically united advantages of each component to boost water-splitting activity, the synergistic effect of heterostructure of bimetallic selenides can efficiently improve the structural stability and promote the generation of active phases during the OER process [31]. However, bimetallic selenides as efficient OER electrocatalysts are still in infancy, and the specific mechanism of the synergistic effect between heterogeneous structures has not been clearly understood [32]. In addition, their catalytic performances have not yet meet industrial demands for electrochemical water-splitting (high current density > 500 mA cm^−2^) [33, 34].

Herein, we developed a superaerophobic 3D heterostructure of bimetallic selenide consisting of crystalline NiSe_2_ and NiFe_2_Se_4_ nanowrinkles on backbones of 3D NiFe alloy, synthesized via a simple one-step thermal selenization procedure. The thickness of NiSe_2_/NiFe_2_Se_4_ nanowrinkles was about 100 nm. On account of the high electronic conductivity and large active surface area, the NiSe_2_/NiFe_2_Se_4_@NiFe heterostructure with high current densities of 500 and 1000 mA cm^−2^ could be output at low potentials of 1.53 and 1.54 V, respectively, which are appreciably superior to almost all previously reported Ni/Fe-based selenides, and even better than commercial Ir/C catalyst. In-situ electrochemical Raman spectroscopy discovered that the formd FeOOH and NiOOH species are the real active phases in NiSe_2_/NiFe_2_Se_4_@NiFe for OER catalysis. In addition, a special “superaerophobic” feature of NiSe_2_/NiFe_2_Se_4_@NiFe enabled an outstanding capability to diminish the negative effects and promote rapid release of in-situ generated O_2_ bubbles during the OER process. Furthermore, the NiSe_2_/NiFe_2_Se_4_@NiFe heterostructure as a bifunctional electrocatalyst exhibited superior electrocatalytic activity for overall-water-splitting in 10.0 M KOH at 60 °C, driven at a low voltage of 2.17 V to achieve 1000 mA cm^−2^.

## Experimental Sections

### Chemicals and Materials

All reagents are analytical grade and used without further purifications. The NiFe alloy, Ni foam (NF), and Fe foam (IF) were purchased from Kunshan Longshengbao Electronic material store. The Se powder, KOH, commercial IrO_2_, ethanol, and acetone were obtained from Alfa Aesar.

#### ***Synthesis of NiSe***_***2***_***/NiFe***_***2***_***Se***_***4***_***@NiFe***

Commercial NiFe alloy was washed in an ultrasonic machine with acetone, hydrochloric acid, ethanol, and deionized water for 10 min, respectively. After the above treatments, two pieces of NiFe alloy (0.25 × 1.5 cm^2^) were thermally selenized at 300 °C for 2 h with 60 mg of Se powder in a vacuum quartz tube. The mass loading of NiSe_2_/NiFe_2_Se_4_ on NiFe alloy was ~ 5.0 mg cm^−2^.

#### ***Synthesis of Ni***_***0.7***_***Fe***_***0.3***_***–Se and Ni***_***0.5***_***Fe***_***0.5***_***–Se***

After the pre-treatments, the Ni_0.7_Fe_0.3_ alloy and Ni_0.5_Fe_0.5_ alloy were thermally selenized at 300 °C for 2 h with 60 mg of Se powder in a vacuum quartz tube, respectively.

#### Synthesis of NF-Se

Commercial Ni foam was washed in an ultrasonic machine with acetone, hydrochloric acid, ethanol, and deionized water for 10 min, respectively. After the above treatments, two pieces of Ni foam were thermally selenized at 300 °C for 2 h with 60 mg of Se powder in a vacuum quartz tube. The mass loading of NF-Se on Ni foam was ~ 4.7 mg cm^−2^.

#### Synthesis of IF-Se

Commercial Fe foam was washed in an ultrasonic machine with acetone, hydrochloric acid, ethanol, and deionized water for 10 min, respectively. After the above treatments, two pieces of Fe foam were thermally selenized at 300 °C for 2 h with 60 mg of Se powder in a vacuum quartz tube. The mass loading of IF-Se on Fe foam was ~ 4.0 mg cm^−2^.

### Characterizations

X-ray diffraction patterns (XRD) were examined on a RIGAKU D/MAX 2550/PC (RIGAKU D/MAX 2550/PC). Field-emission scanning electron microscopy (FESEM) images were investigated on a SU-8010 at an acceleration voltage of 5 kV. Transmission electron microscopy (TEM) images, high-resolution TEM (HRTEM) images, selected-area electron diffraction (SAED) patterns were obtained on a JEM-2100 electron microscope (HRTEM, JEM-2100, 200 kV) equipped with an energy dispersive X-ray spectrometer, operating at 120 kV. Raman spectra were obtained by a Raman scattering spectroscopy system, excited with a 534 nm diode laser. The surface elemental information was obtained by X-ray photoelectron spectroscopy performed on the RIGAKU D/MAX 2550/PC (RIGAKU D/MAX 2550/PC). Contact angles were analyzed via an OCA20 machine (Data-Physics, Germany) at room temperature. The atom ratio of Fe and Ni were analyzed via X-ray fluorescence spectrometry (Rigaku, ZSX Primus II) at room temperature.

### Electrochemical Measurements

All measured potentials in this work were reported versus reversible hydrogen electrode (RHE) according to the equation: *E*_RHE_ = *E*_applied_ + 0.197 + 0.059 pH, where the *E*_applied_ is the applied potential. Linear sweep voltammetry (LSV) curves were recorded at a voltage range of 1.2–0 V with a scan rate of 5 mV s^−1^. In OER performance test, all polarization curves were with iR compensation in this work unless otherwise noted. The long-term durability test was performed using a chronopotentionmetry method at a constant current density. The *C*_dl_ values of the as-prepared working electrodes were determined from the cyclic voltammogram (CV) in the double layer region (without Faradaic processes) at different scan rates.

## Results and Discussion

### Structural Characterizations of NiSe_2_/NiFe_2_Se_4_@NiFe

**Fig. 1:**
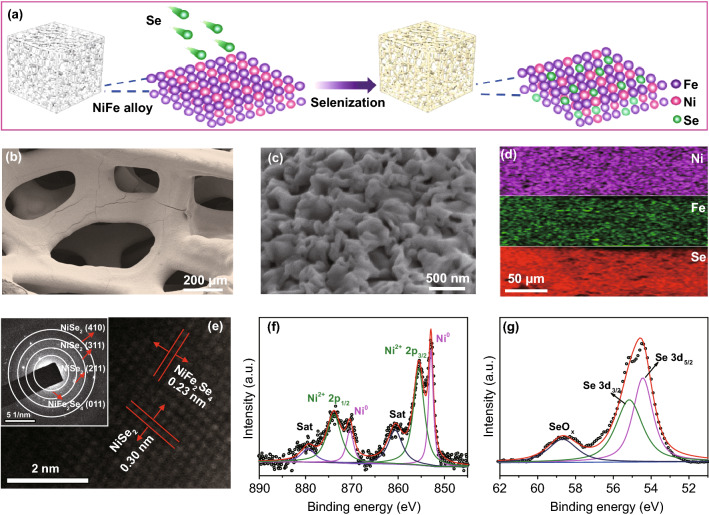
**a** Schematic illustration for one-step fabrication strategy of NiSe_2_/NiFe_2_Se_4_@NiFe. **b**, **c** FESEM images of NiSe_2_/NiFe_2_Se_4_@NiFe. **d** EDX elemental mappings images of Ni, Fe, and Se elements in NiSe_2_/NiFe_2_Se_4_@NiFe. **e** HRTEM image of NiSe_2_/NiFe_2_Se_4_@NiFe; inset: corresponding SAED pattern. High-resolution XPS spectra of **f** Ni 2*p* and **g** Se 3d for NiSe_2_/NiFe_2_Se_4_@NiFe

Figure 1a illustrates a facile thermal selenization process of 3D NiSe_2_/NiFe_2_Se_4_@NiFe synthesis. The NiFe alloy was annealed at 300 °C for 2 h under a selenium vapor atmosphere to obtain the NiSe_2_/NiFe_2_Se_4_@NiFe. We systematically investigated the effects of different selenium contents and selenization temperatures on OER capacity, and the optimal amount of selenium powder was 60 mg and annealing temperature was 300 °C (Figs. S1-S3). FESEM image reveal that the spatial skeleton morphology of the 3D NiSe_2_/NiFe_2_Se_4_@NiFe remained after the thermal selenization process (Fig. 1b, c) [35]. The NiSe_2_/NiFe_2_Se_4_@NiFe heterostructure was consisted of numerous nanowrinkles with a thickness of ~ 100 nm. The corresponding energy-dispersive X-ray spectroscopy (EDX) element mapping images showed the uniform coverage of Se, Fe, and Ni elements on the surface of 3D NiFe alloy (Fig. 1d). TEM and HRTEM images (Fig. 1e and Figs. S4, S5) of NiSe_2_/NiFe_2_Se_4_@NiFe displayed that the characteristic spacings of 0.30 and 0.27 nm are attributed to the (200) and (210) planes of NiSe_2_, while the characteristic distances of 0.34 and 0.23 nm are corresponded to the (011) and (211) planes of NiFe_2_Se_4_, respectively. Further, an obvious boundary of the crystal surface between NiSe_2_ and NiFe_2_Se_4_ was clearly observed (Fig. S6), successfully revealing the formed heterostructure of NiSe_2_/NiFe_2_Se_4_@NiFe. SAED pattern showed the well-crystallized of NiSe_2_ and NiFe_2_Se_4_ in NiSe_2_/NiFe_2_Se_4_@NiFe (inset of Fig. 1e) [36].

XRD of NiSe_2_/NiFe_2_Se_4_@NiFe (Fig. S7) showed the characteristic diffraction peaks of NiSe_2_ (JPCDS No. 11-0552) and NiFe_2_Se_4_ (JPCDS No. 065-2338) [37]. The ratio of NiSe_2_ and NiFe_2_Se_4_ in the NiSe_2_/NiFe_2_Se_4_@NiFe was determined to be ~ 2.58 according to inductively coupled X-ray fluorescence spectrometry. X-ray photoelectron spectroscopy (XPS) revealed the co-habiting of Ni, Fe, and Se elements in the NiSe_2_/NiFe_2_Se_4_ (Fig. S8). The high-resolution Ni 2*p* XPS spectra of NiSe_2_/NiFe_2_Se_4_@NiFe heterostructure displayed two principal peaks located at 873.8 and 855.5 eV, which are corresponding to the Ni^2+^ 2*p*_1/2_ and Ni^2+^ 2*p*_3/2_ (Fig. 1f), associated with two satellite peaks. Relative to the pure NiSe_2_ [29, 38], a slight shift of the principal peaks toward the higher binding energies was observed for NiSe_2_/NiFe_2_Se_4_@NiFe, which could be attributed to strongly coupled effects between NiSe_2_ and NiFe_2_Se_4_ in the heterostructure [39]. Furthermore, the binding energies centered at 852.9 and 870.3 eV are corresponding to metallic Ni from the NiFe alloy. The high-resolution Se 3*d* XPS spectra of NiSe_2_/NiFe_2_Se_4_@NiFe exhibited three contributions, including two Se 3*d*_5/2_ and Se 3*d*_3/2_ peaks located at 54.5 and 55.2 eV, respectively, and one low and wide peak located at 58.6 eV of SeO_*x*_ species (Fig. 1g) [40, 41]. It is important that, the Se 3*d* peaks at 54.5 and 55.2 eV are separately located between 54.0 eV for Se^2−^ and 54.7 eV for Se_2_^2−^ as well as between 54.9 eV for Se^2−^ and 55.6 eV for Se_2_^2−^, suggesting the co-existence of NiSe_2_ and NiFe_2_Se_4_ [42].

### Electrocatalytic OER Performance

**Fig. 2:**
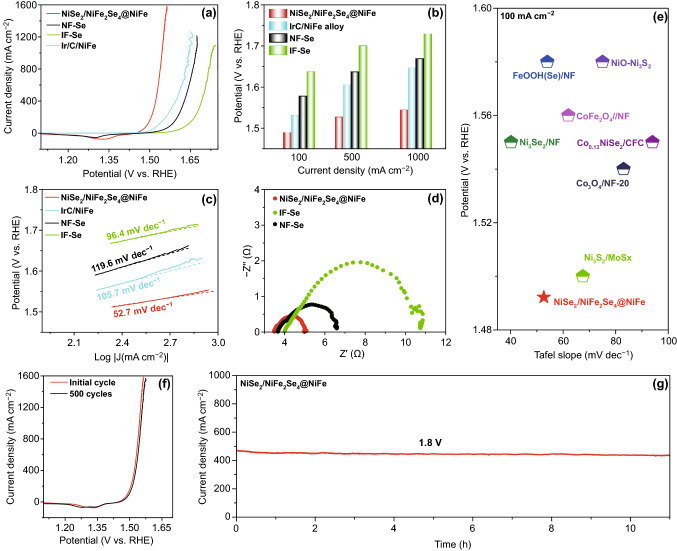
**a** Polarization curves of NiSe_2_/NiFe_2_Se_4_@NiFe, NF-Se, IF-Se, and Ir/C/NiFe with iR compensation. **b** Comparison of potentials required at 100, 500, and 1000 mA cm^−2^ for NiSe_2_/NiFe_2_Se_4_@NiFe, NF-Se, IF-Se, and Ir/C/NiFe. **c** Tafel plots of NiSe_2_/NiFe_2_Se_4_@NiFe, NF-Se, IF-Se, and Ir/C/NiFe. **d** Nyquist plots of NiSe_2_/NiFe_2_Se_4_@NiFe, NF-Se, and IF-Se. **e** Comparison of Tafel slopes and potentials required for 100 mA cm^−2^. **f** Polarization curves of NiSe_2_/NiFe_2_Se_4_@NiFe before and after 500 cycles with iR compensation. **g** Chronoamperometry curve with the NiSe_2_/NiFe_2_Se_4_@NiFe as electrode at 1.8 V without iR compensation. Electrolyte: 1.0 M KOH

The OER polarization curve of NiSe_2_/NiFe_2_Se_4_@NiFe was first determined in 1.0 M KOH electrolyte. In comparison, the control samples of Ni foam@60 mg Se 300 °C (NF-Se), Fe foam@60 mg Se 300 °C (IF-Se), and Ir/C/NiFe were also tested. As shown in Fig. 2a, b, the NiSe_2_/NiFe_2_Se_4_@NiFe exhibited admirable electrocatalytic activity with smaller potentials of 1.49, 1.53, and 1.54 V at current densities of 100, 500, and 1000 mA cm^−2^, compared with those of NF-Se and IF-Se. Significantly, the potential needed to drive a large current density up to 1500 mA cm^−2^ was only 1.56 V, which makes the NiSe_2_/NiFe_2_Se_4_@NiFe up-and-coming OER electrocatalyst towards industrial applications in alkaline water splitting. Meanwhile, the OER Faradaic efficiency of NiSe_2_/NiFe_2_Se_4_@NiFe was calculated to be ∼ 100% (Fig. S9).

Comparison of the OER performances among NiSe_2_/NiFe_2_Se_4_@NiFe, NF-Se, and IF-Se demonstrated the positive effects of the synergistic effect between NiSe_2_ and NiFe_2_Se_4_ components, which contributed to the superior OER activities of the NiSe_2_/NiFe_2_Se_4_@NiFe. Apparently, the OER catalytic activity of NiSe_2_/NiFe_2_Se_4_@NiFe significantly surpassed that of the benchmark Ir/C/NiFe, which showed high potentials of 1.53, 1.60, and 1.65 V at 100, 500, and 1000 mA cm^−2^, respectively. The Tafel slope of NiSe_2_/NiFe_2_Se_4_@NiFe was 52.7 dec^−1^ (Fig. 2c), which was much smaller than the Tafel slopes of NF-Se (105.7 dec^−1^), Fe–Se (119.6 dec^−1^), and Ir/C/NiFe (96.4 mV dec^−1^), indicating a rapid reaction kinetic of the NiSe_2_/NiFe_2_Se_4_@NiFe. The OER kinetics of NiSe_2_/NiFe_2_Se_4_@NiFe was further investigated by electrochemical impedance spectroscopy (EIS), and the Nyquist plots of the NiSe_2_/NiFe_2_Se_4_@NiFe showed the much lower charge-transfer impedance as compared with that of NF-Se and IF-Se (Fig. 2d), supporting the fast electron transfer ability of NiSe_2_/NiFe_2_Se_4_@NiFe. The potential at 100 mA cm^−2^ and corresponding Tafel slope of NiSe_2_/NiFe_2_Se_4_@NiFe were appreciably superior than that of those previously reported Ni/Fe-based selenides and other non-precious OER electrocatalysts in 1.0 M KOH (Fig. 2e and Table S1) [6, 40, 43–47].

Figure S10 shows a multi-step chronopotentiometric curve of NiSe_2_/NiFe_2_Se_4_@NiFe, in which the starting current density was 380 mA cm^−2^ at 1.67 V, and remained unchanged for the rest 100 s; the other four steps also exhibited parallel results up to 1180 mA cm^−2^, suggesting the remarkable mass transport properties and mechanical toughness of NiSe_2_/NiFe_2_Se_4_@NiFe [48, 49]. As the durability is another effective standard to assess the electrocatalytic ability of NiSe_2_/NiFe_2_Se_4_@NiFe, continuous electrochemical cycling tests were performed for 500 cycles. In Fig. 2f, the NiSe_2_/NiFe_2_Se_4_@NiFe exhibited permanent stability with a minor current loss at the end of cycling. Further, the NiSe_2_/NiFe_2_Se_4_@NiFe also owned long-term durability with an insignificant recession during consecutive current output at 500 mA cm^−2^ over 11 h (Fig. 2 g).

**Fig. 3:**
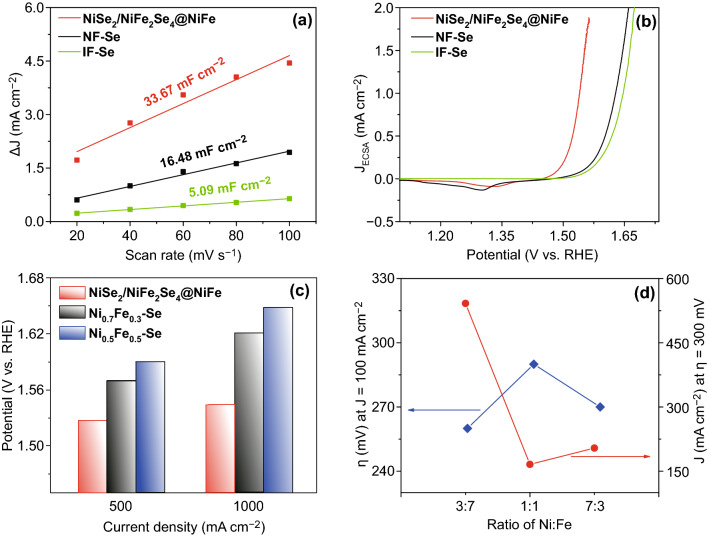
**a** ECSAs and **b** ECSA-normalized polarization curves of NiSe_2_/NiFe_2_Se_4_@NiFe, NF-Se, and IF-Se. **c** Comparison of potentials required at 500 and 1000 mA cm^−2^ for NiSe_2_/NiFe_2_Se_4_@NiFe, Ni_0.7_Fe_0.3_–Se, and Ni_0.5_Fe_0.5_–Se. **d** OER activities of NiSe_2_/NiFe_2_Se_4_@NiFe, Ni_0.7_Fe_0.3_–Se, and Ni_0.5_Fe_0.5_–Se at 100 mA cm^−2^ and 300 mV

In order to identify the synergistic effect of bimetallic selenide heterostructure towards the extrusive OER performance, we measured double-layer capacitance (*C*_dl_) to evaluate the electrochemical surface area (ECSA) of 3D NiSe_2_/NiFe_2_Se_4_@NiFe. As shown in Fig. 3a and Fig. S11, the *C*_dl_ of 33.67 mF cm^−2^ for NiSe_2_/NiFe_2_Se_4_@NiFe was much higher than 16.48 mF cm^−2^ for NF-Se and 5.09 mF cm^−2^ for IF-Se, illustrating that the NiSe_2_/NiFe_2_Se_4_@NiFe possessed an extraordinary activity with larger ECSA and more accessible active sites as compared with the NF-Se and IF–Se. For clarification of the inherent OER activity of 3D NiSe_2_/NiFe_2_Se_4_@NiFe, the polarization curve of the heterostructure electrode was further normalized by ECSA (Fig. 3b), and the results displayed that the intrinsic activity of NiSe_2_/NiFe_2_Se_4_@NiFe was still much higher than that of NF-Se and IF-Se [50, 51]. In order to further investigate the influence of Ni: Fe ratios, we additionally constructed other catalytic materials of Ni_0.5_Fe_0.5_@60 mg Se 300 °C (Ni_0.5_Fe_0.5_–Se) and Ni_0.7_Fe_0.3_@60 mg Se 300 °C (Ni_0.7_Fe_0.3_–Se) with different Ni/Fe proportions. As shown in Fig. 3c, d and Fig. S12, the OER performances of the NiSe_2_/NiFe_2_Se_4_@NiFe, Ni_0.5_Fe_0.5_–Se, and Ni_0.7_Fe_0.3_–Se displayed that the potentials of Ni_0.5_Fe_0.5_–Se and Ni_0.7_Fe_0.3_–Se were 1.59 and 1.57 V at 500 mA cm^−2^, which are significantly larger than that of the NiSe_2_/NiFe_2_Se_4_@NiFe (1.53 V at 500 mA cm^−2^). Also, when the overpotential was fixed at 300 mV, the current densities of both Ni_0.5_Fe_0.5_–Se and Ni_0.7_Fe_0.3_–Se were 166 and 204 mA cm^−2^ (Fig. 3d), extremely smaller than that of NiSe_2_/NiFe_2_Se_4_@NiFe (542 mA cm^−2^). These results indicated that the NiSe_2_/NiFe_2_Se_4_@NiFe with a Ni/Fe ratio of 3:7 possessed the optimized OER activity in comparison with Ni_0.5_Fe_0.5_–Se and Ni_0.7_Fe_0.3_–Se [52].

### Identifying Active Phase

**Fig. 4:**
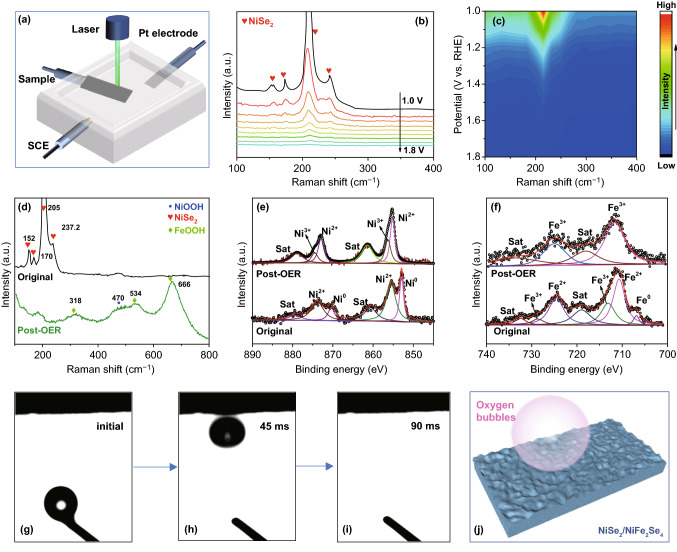
**a** Digital images of in-situ electrochemical Raman spectroscopy for NiSe_2_/NiFe_2_Se_4_@NiFe during OER process. **b** In-situ Raman spectra and **c** corresponding contour plots of NiSe_2_/NiFe_2_Se_4_@NiFe at various potentials. **d** Raman spectra of NiSe_2_/NiFe_2_Se_4_@NiFe before and after OER tests. **e**, **f** High-resolution Ni 2*p* and Fe 2*p* XPS spectra of NiSe_2_/NiFe_2_Se_4_@NiFe before and after OER tests. **g**–**i** Digital images of O_2_ bubbles on NiSe_2_/NiFe_2_Se_4_@NiFe. **j** Schematic illustration of the adhesion behavior for O_2_ bubbles on NiSe_2_/NiFe_2_Se_4_@NiFe

The structural change of NiSe_2_/NiFe_2_Se_4_@NiFe during the OER process was analyzed by in-situ electrochemical Raman spectroscopy at different applied potentials in 1.0 M KOH (Fig. 4a). At a voltage of 1.0 V, the Raman peaks of NiSe_2_/NiFe_2_Se_4_@NiFe located at 152, 170, 205, and 237 cm^−1^ could be assigned to NiSe_2_ (Fig. 4b, c); it was clearly seen that the Raman peaks of NiSe_2_ gradually weakened with the increased voltage. After the OER tests (Fig. 4d), no Raman peaks of NiSe_2_ were detected, but new distinctive peaks associated with the unique Raman features of amorphous FeOOH and NiOOH species could be observed, which indicated that the amorphous FeOOH and NiOOH phases are the catalytically active phases of NiSe_2_/NiFe_2_Se_4_@NiFe during the OER process [39, 53]. Such features are also consistence well with post-HRTEM observations (Fig. S13). The chemical valence states of NiSe_2_/NiFe_2_Se_4_@NiFe before and after OER tests were measured by XPS spectra. After 11 h OER stability testing, in the high-resolution XPS spectra of Ni 2*p* (Fig. 4e), the binding energy of metallic Ni disappeared, and the binding energies of Ni^3+^ 2*p*_3/2_ and Ni^3+^ 2*p*_1/2_ emerged and located at 856.4 and 874.5 eV, which indicated the oxidation of Ni^2+^ to Ni^3+^ [54]. For the high-resolution XPS spectra of Fe 2*p* (Fig. 4f), four main peaks located at 710.7, 713.2, 724.6, and 728.7 eV were corresponded well with Fe^2+^ 2*p*_3/2_, Fe^3+^ 2*p*_3/2_, Fe^2+^ 2*p*_1/2_, and Fe^3+^ 2*p*_1/2_, respectively. After the OER tests, two new Fe 2*p*_3/2_ peaks appeared at 711.7 and 725.1 eV, respectively, which are the characteristic binding energies of Fe^3+^ in FeOOH [1]. These results demonstrated that the in-situ derived amorphous FeOOH and NiOOH phases serve as OER active centers in NiSe_2_/NiFe_2_Se_4_@NiFe during the OER process, which was in accord with the Raman results. Based on the above results, a possible mechanism of the OER electrocatalysis in alkaline electrolyte has been considerate as follows [43, 55, 56]:

1 $${\text{M}}_{{{\text{cat}}}} + {\text{OH}}^{ - } \to {\text{M}}_{{{\text{cat}}}} {\text{OH}}_{{{\text{ad}}}} + {\text{e}}^{ - }$$

2 $${\text{M}}_{{{\text{cat}}}} {\text{OH}}_{{{\text{ad}}}} + {\text{OH}}^{ - } \to {\text{M}}_{{{\text{cat}}}} {\text{O}}_{{{\text{ad}}}} + {\text{H}}_{2} {\text{O}} + {\text{e}}^{ - }$$

3 $${\text{M}}_{{{\text{cat}}}} {\text{O}}_{{{\text{ad}}}} + {\text{OH}}^{ - } \to {\text{M}}_{{{\text{cat}}}} {\text{OOH}}_{{{\text{ad}}}} + {\text{e}}^{ - }$$

4 $${\text{M}}_{{{\text{cat}}}} {\text{OOH}}_{{{\text{ad}}}} + {\text{OH}}^{ - } \to {\text{M}}_{{{\text{cat}}}} + {\text{O}}_{2} + {\text{H}}_{2} {\text{O}} + {\text{e}}^{ - }$$

where the NiOOH and FeOOH species were firstly formed on the surface of NiSe_2_/NiFe_2_Se_4_@NiFe accessed by OH^−^ in alkaline electrolyte. Then, the formed NiOOH and FeOOH phases were further combined with the OH^−^ to generate O_2_ under OER conditions (M = Ni, Fe) [57–60].

Considering that the O_2_ bubbles generated under the harsh electrochemical conditions tend to decrease the surface roughness and limit electron transfer, the contact wetting angle of NiSe_2_/NiFe_2_Se_4_@NiFe was measured (Fig. 4g–j). The results showed that the generated O_2_ bubbles are separated in an ultra-fast speed from the surface, indicating a “superaerophobic” feature. The unique “superaerophobic” structure of NiSe_2_/NiFe_2_Se_4_@NiFe could deliver a huge potential to release the in-situ generated O_2_ bubbles and avoid the bubbles to be detented during the OER process, thus retaining the original catalytic sites of NiSe_2_/NiFe_2_Se_4_@NiFe. Therefore, besides the FeOOH and NiOOH active phases, the unique “superaerophobic” property of NiSe_2_/NiFe_2_Se_4_@NiFe that can expel the in-situ generated O_2_ bubbles also make a contribution to the high-efficient OER activity and excellent stability at high current densities [61].

### Overall-Water-Splitting Performance

**Fig. 5:**
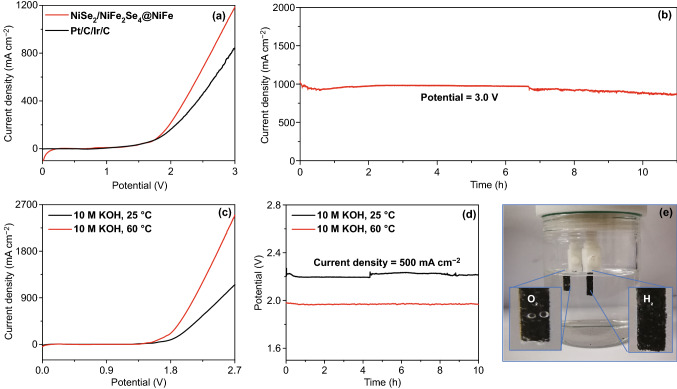
**a** Polarization curves of bifunctional NiSe_2_/NiFe_2_Se_4_@NiFe and Pt/C-Ir/C for overall-water-splitting. **b** Chronoamperometric measurement at 3.0 V across NiSe_2_/NiFe_2_Se_4_@NiFe without iR compensation. **c** Polarization curves of bifunctional NiSe_2_/NiFe_2_Se_4_@NiFe for overall-water-splitting in 10.0 M KOH at 25 and 60 °C. **d** Chronopotentiometric curves with bifunctional NiSe_2_/NiFe_2_Se_4_@NiFe in 10.0 M KOH at 25 and 60 °C at 500 mA cm^−2^ without iR compensation. **e** Digital image of the generated H_2_ and O_2_ gas on NiSe_2_/NiFe_2_Se_4_@NiFe

Based on the outstanding OER performances, the 3D NiSe_2_/NiFe_2_Se_4_@NiFe were applied as both anode and cathode for the testing of overall-water-splitting. The NiSe_2_/NiFe_2_Se_4_@NiFe exhibited a bifunctional electrocatalytic performance in overall-water-splitting, which only needed cell voltages of 2.32 and 2.82 V to reach high current densities of 500 and 1000 mA cm^−2^ in 1.0 M KOH, respectively. Notably, such a high overall-water-splitting performance for NiSe_2_/NiFe_2_Se_4_@NiFe was even superior to that of precious metal catalysts of Ir/C anode and Pt/C cathode with larger cell voltages of 2.56 and > 3.0 V at 500 and 1000 mA cm^−2^ (Fig. 5a), respectively. Furthermore, the long-term stability of NiSe_2_/NiFe_2_Se_4_@NiFe in electrochemical overall-water-splitting was confirmed with a high current density at 1000 mA cm^−2^ for > 10 h (Fig. 5b). As required for an industrial use, we further made an alkaline electrolyzer for overall-water-splitting using the bifunctional NiSe_2_/NiFe_2_Se_4_@NiFe in 10.0 M KOH at 25 and 60 °C. As shown in Fig. 5c, the NiSe_2_/NiFe_2_Se_4_@NiFe delivered the higher overall-water-splitting performance at 60 °C (2.17 V at 1000 mA cm^−2^) than that at 25 °C [62]. Furthermore, the NiSe_2_/NiFe_2_Se_4_@NiFe sustained durable stability with a low voltage of 1.96 V to achieve 500 mA cm^−2^ in 10.0 M KOH at 60 °C after chronoamperometry testing (Fig. 5d). During the testing process, the generated O_2_ and H_2_ bubbles are in-situ produced on the bifunctional NiSe_2_/NiFe_2_Se_4_@NiFe (Fig. 5e), evidencing the favorable overall-water-splitting performance. These results suggested that the NiSe_2_/NiFe_2_Se_4_@NiFe could satisfy the industrial criteria for overall-water-splitting electrocatalysis.

## Conclusions

A novel superaerophobic 3D NiSe_2_/NiFe_2_Se_4_@NiFe heterostructure composing of NiSe_2_ and NiFe_2_Se_4_ nanowrinkles was developed by a thermal selenization procedure. The NiSe_2_/NiFe_2_Se_4_@NiFe showed excellent OER performance evidenced by outputting high current densities of 500 and 1000 mA cm^−2^ at low potentials of 1.53 and 1.54 V under alkaline condition, respectively, which are superior to those of most previously reported Ni/Fe-based selenides, even outperforming the commercial Ir/C. The excellent OER performance of NiSe_2_/NiFe_2_Se_4_@NiFe to a large extent was due to the large active surface area and high electronic conductivity. The in-situ conversion-derived FeOOH and NiOOH species from the NiSe_2_/NiFe_2_Se_4_@NiFe are intrinsic active sites for the OER catalysis. The unique “superaerophobic” structure of NiSe_2_/NiFe_2_Se_4_@NiFe further promoted the rapid release of in-situ formed O_2_ bubbles in a superfast speed. The NiSe_2_/NiFe_2_Se_4_@NiFe heterostructure required a low voltage of 2.17 V to attain 1000 mA cm^−2^ in 10.0 M KOH electrolyte for overall-water-splitting at 60 °C meeting the requirement for industrial use. Therefore, the NiSe_2_/NiFe_2_Se_4_@NiFe heterostructure presented in this work may provide a promising way to synthesize superaerophobic bimetallic selenides towards the practical applications for clean hydrogen production, as well as the electrochemical CO_2_ reduction, O_2_ reduction, and N_2_ reduction reactions.

## Electronic supplementary material

Below is the link to the electronic supplementary material.

Supplementary file1 (PDF 1046 kb)
