# Supplementary material for: A Superaerophobic Bimetallic Selenides Heterostructure for Efficient Industrial-Level Oxygen Evolution at Ultra-High Current Densities
Source: Nanomicro Lett. 2020 May 2;12:104. doi: 10.1007/s40820-020-00442-0 (PMC7770871; doi:10.1007/s40820-020-00442-0)
Supplement: Supplementary file 1 — Supplementary file1 (PDF 1046 kb) [file 40820_2020_442_MOESM1_ESM.pdf]

Supporting Information for

A Superaerophobic Bimetallic Selenides Heterostructure for Efficient Industrial-level Oxygen Evolution at Ultra-High Current Densities

Jiaxin Yuan[1], Xiaodi Cheng[1], Hanqing Wang[1, 6], Chaojun Lei[1], Sameer Pardiwala[1], Bin Yang[1], Zhongjian Li[1], Qinghua Zhang[4], Lecheng Lei[1], Shaobin Wang[5, *], Yang Hou[1, 2, 3, *]

[1]Key Laboratory of Biomass Chemical Engineering of Ministry of Education, College of Chemical and Biological Engineering, Zhejiang University, Hangzhou 310027, People's Republic of China

[2]Institute of Zhejiang University - Quzhou, Quzhou 324000, People's Republic of China

[3]Ningbo Research Institute, Zhejiang University, Ningbo 315100, People's Republic of China

[4]Zhejiang Provincial Key Laboratory of Advanced Chemical Engineering Manufacture Technology, College of Chemical and Biological Engineering, Zhejiang University, Hangzhou 310027, People's Republic of China

[5]School of Chemical Engineering and Advanced Materials, The University of Adelaide, Adelaide 5005, SA, Australia

[6] Zhejiang Province Hangzhou No. 14 High School, Hangzhou 310027, People's Republic of China

*Corresponding authors. E-mail: yhou@zju.edu.cn (Yang Hou); shaobin.wang@adelaide.edu.au (Shaobin Wang)

S1 Synthesis of Ir/C/NiFe and Pt/C/NiFe

A mixture of 9.0 mg of Ir/C, 810 μL of Nafion (5%), and 90 μL of ethanol was ultrasonicated for 30 min, and then oscillated to obtain uniform dispersion. After the Ir/C dispersion dropped onto the treated NiFe alloy, the Ir/C/NiFe was gradually dried in a fume hood. The loading amount of Ir/C was ~5.0 mg cm^{-2}. The similar procedure was used to prepare Pt/C/NiFe.

S2 Electrochemical Measurements

Electrochemical activity tests were operated in a traditional three-electrode system at room temperature, using a carbon rod and an Ag/AgCl electrode as the counter and reference electrodes, respectively. The $NiSe_2/NiFe_2Se_4$@NiFe was used as the working electrode and the electrolyte was 1.0 M KOH solution. During the controlled

experiments, the evenly dispersed commercial Pt/C and Ir/C samples were loaded onto the surface of clean NiFe alloy as the working electrodes.

The OER curves were normalized by electrochemical surface area (ECSA) to eliminate the influence of the ECSA on the performance comparisons. The ECSA-normalized current density for as-prepared samples was calculated as below: ECSA-normalized current density = current density × C_s/C_{dl}

where C_s is the specific capacitance, and 0.04 mF cm^{-2} is adopted as the value of C_s based on previously reported OER catalysts in alkaline solution [S1].

Calculation for Faradaic Efficiency: Electrolysis was performed by quantitative gas chromatography (GC) under a constant potential (1.55, 1.60, 1.65, 1.70, and 1.75 V) running for 20 min in a custom-built H-type cell in which the column Pt electrode is placed in one compartment while the Ag/AgCl electrode and $NiSe_2/NiFe_2Se_4$@NiFe electrode are placed in another. The product was subsequently detected by a thermal conductivity detector (TCD) in quantitative GC equipment. Atmospheric N_2 was used as an internal standard. The Faradaic efficiency was calculated by Eq. S1:

$$\text{Faradic efficiency } \% = 4n\text{F/Q} \qquad \text{(S1)}$$

Where F and n are the Faraday constant and the amount of produced O_2, respectively; Q is the total amount of charge flowed past the electrochemical cell [S2, S3].

Overall-water-splitting measurements were performed in a two-electrode system consisting of $NiSe_2/NiFe_2Se_4$@NiFe as anode and cathode. The LSV curve for overall-water-splitting was recorded at a rate of 5 mV s^{-1} in 1.0 M KOH.

The iR compensation was executed based on Eq. S2:

$$E = E_0 - iR \qquad \text{(S2)}$$

where E (unit V) is the potential after iR compensation at the current of i (unit A), E_0 (unit V) is the potential from the polarization curve, i is the current at E_0 from the polarization curve, and R (unit ohm) is the resistance obtained from the EIS result.

S3 Formation Mechanism

The specific reaction mechanism of the formation of $NiSe_2/NiFe_2Se_4$@NiFe heterostructure was provided. During the synthesis process, two main oxidation and reduction reactions occurred under vacuum condition (Eqs. S3 and S4) as below:

$$Ni^0 + Se^0 \rightarrow NiSe_2 \text{ (300 °C)} \qquad \text{(S3)}$$

$$Ni^0 + Se^0 + Fe^0 \rightarrow NiFe_2Se_4 \text{ (300 °C)} \qquad \text{(S4)}$$

From Eqs. S3 and S4, the $NiSe_2$ and $NiFe_2Se_4$ could be generated by the thermal selenization treatment of Ni^0 and Fe^0 species (e.g. NiFe alloy), which is consistent well with the previously reported results [S4, S5].

S4 Supplementary Figures

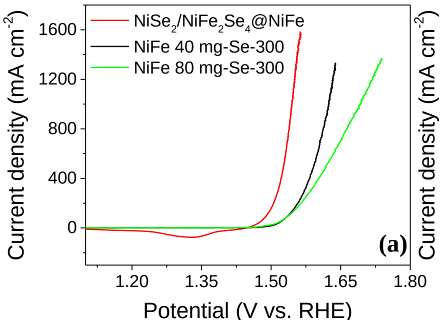

Fig. S1 (**a**) Polarization curves of $NiSe_2/NiFe_2Se_4$@NiFe, NiFe 40 mg Se-300, and NiFe 80 mg Se-300. (**b**) Polarization curves of $NiSe_2/NiFe_2Se_4$@NiFe, NiFe-Se-200, and NiFe-Se-400

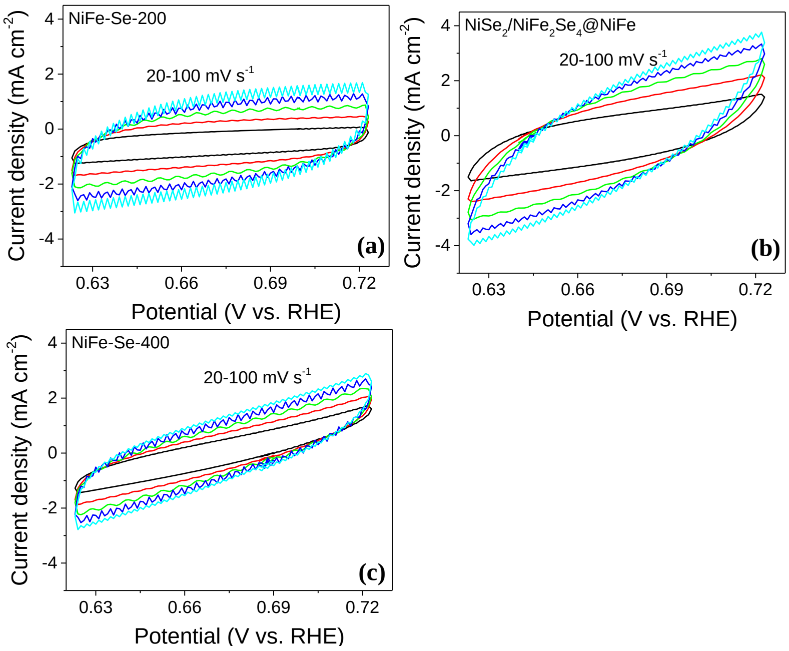

Fig. S2 ECSAs of NiFe-Se-200 (**a**), $NiSe_2/NiFe_2Se_4$@NiFe (**b**), and NiFe-Se-400 (**c**)

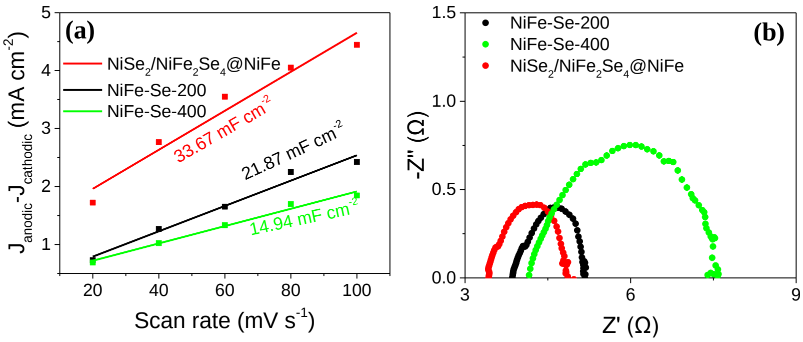

Fig. S3 C_{dl} (**a**) and Nyquist plots (**b**) of NiFe-Se-200, $NiSe_2/NiFe_2Se_4$@NiFe, and NiFe-Se-400

The electrochemical double layer capacitances (C_{dl}) showed that the C_{dl} of 33.67 mF cm^{-2} for $NiSe_2/NiFe_2Se_4$@NiFe was higher than the 21.87 mF cm^{-2} for NiFe-Se-200 and 14.94 mF cm^{-2} for NiFe-Se-400, illustrating that the $NiSe_2/NiFe_2Se_4$@NiFe possessed extraordinary OER activity with more active surface area compared with the NiF-Se-200 and NiFe-Se-400. The electrochemical impedance spectroscopy (EIS) showed a much smaller charge-transfer resistance for $NiSe_2/NiFe_2Se_4$@NiFe as compared with that of NiFe-Se-200 and NiFe-Se-400, suggesting a fast electron transfer ability in $NiSe_2/NiFe_2Se_4$@NiFe.

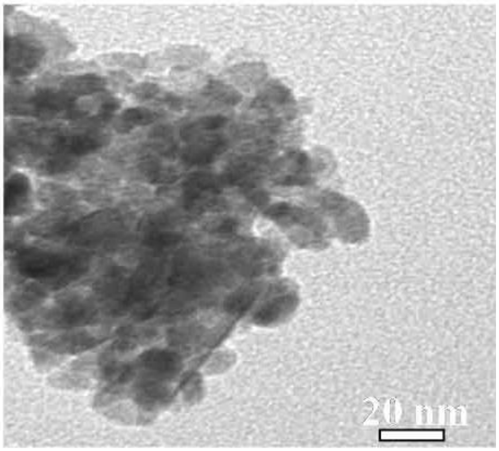

Fig. S4 TEM image of $NiSe_2/NiFe_2Se_4$@NiFe

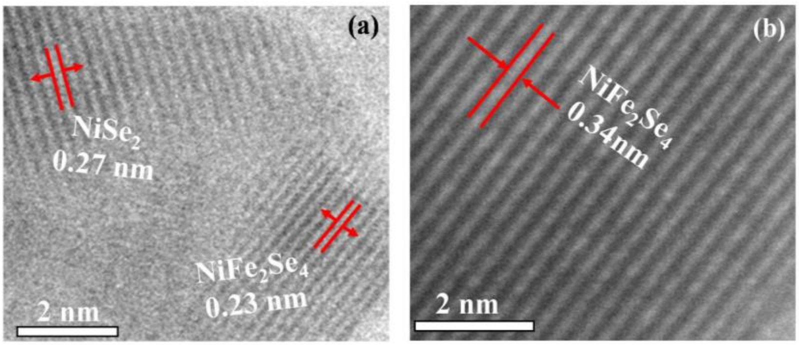

Fig. S5 (a-b) HRTEM images of $NiSe_2/NiFe_2Se_4$@NiFe

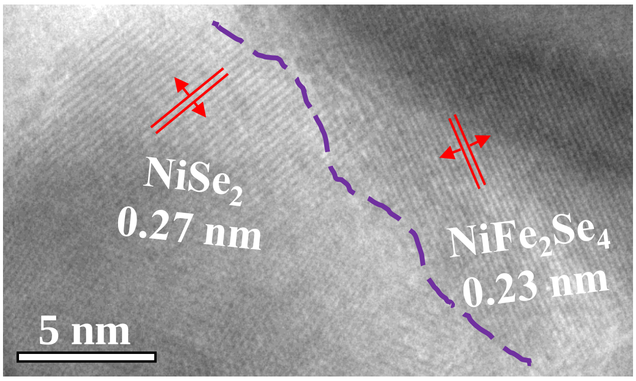

Fig. S6 HRTEM image of $NiSe_2/NiFe_2Se_4$@NiFe

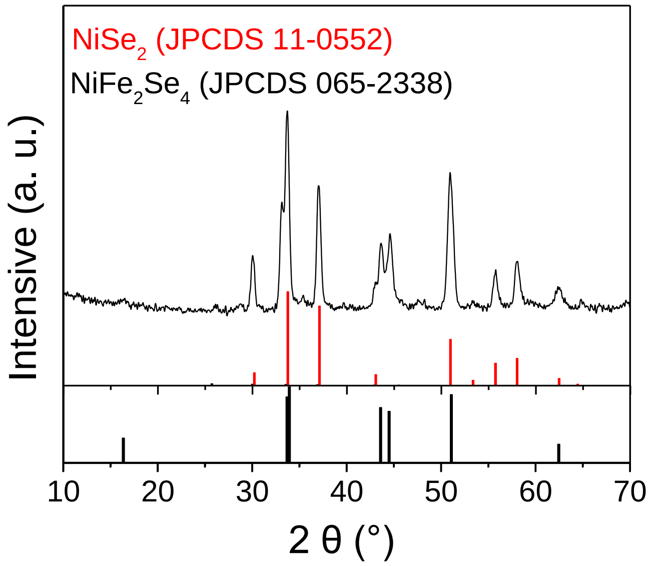

Fig. S7 XRD pattern of $NiSe_2/NiFe_2Se_4$@NiFe

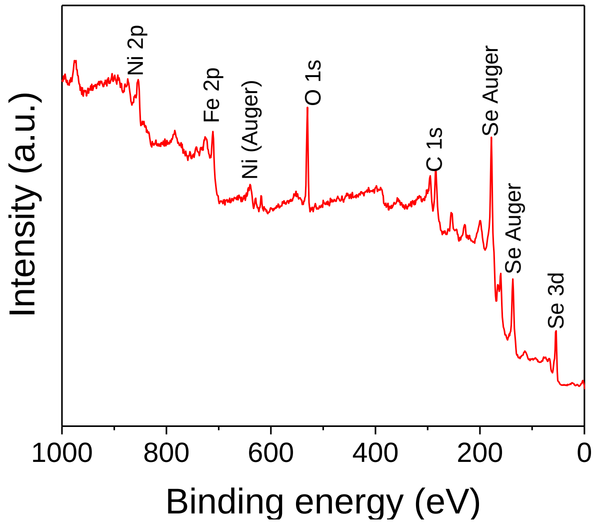

Fig. S8 XPS survey spectrum of $NiSe_2/NiFe_2Se_4$@NiFe

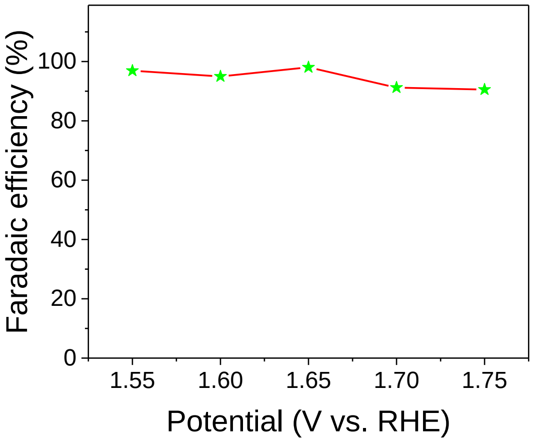

Fig. S9 Faradaic efficiency of $NiSe_2/NiFe_2Se_4$@NiFe for OER

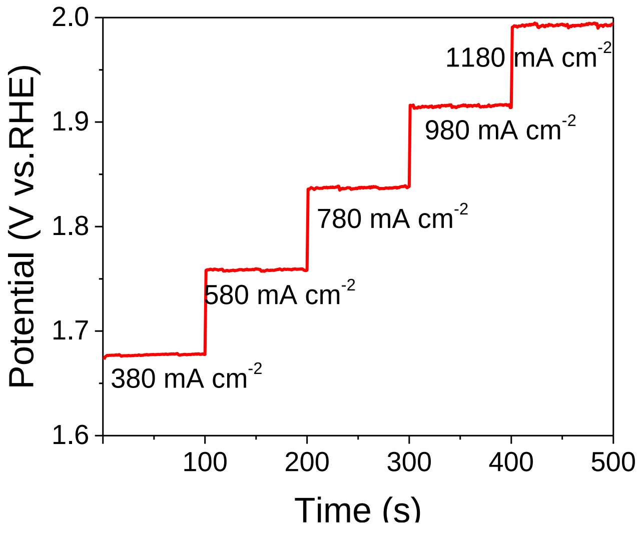

Fig. S10 Multi-step chronopotentiometric curve for $NiSe_2/NiFe_2Se_4$@NiFe

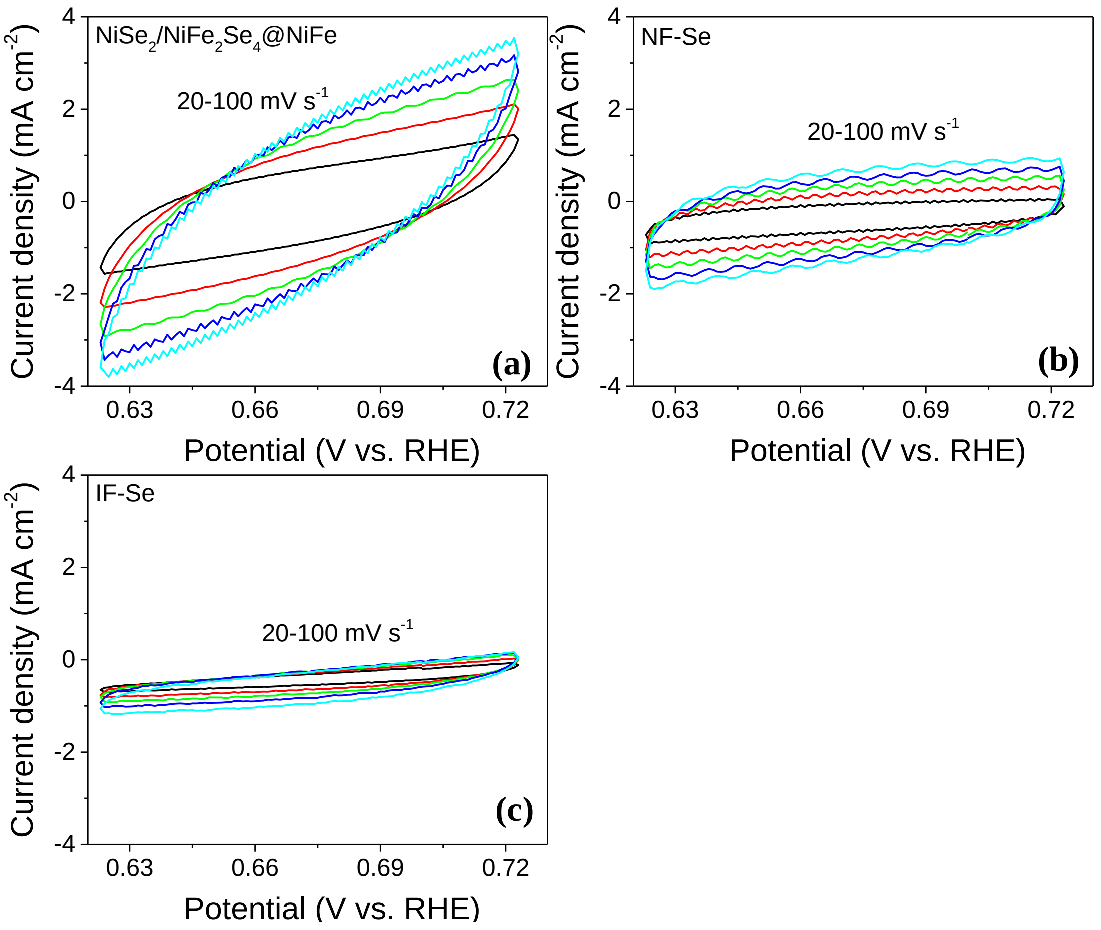

Fig. S11 ECSAs of (**a**) $NiSe_2/NiFe_2Se_4$@NiFe, (**b**) NF-Se, and (**c**) IF-Se

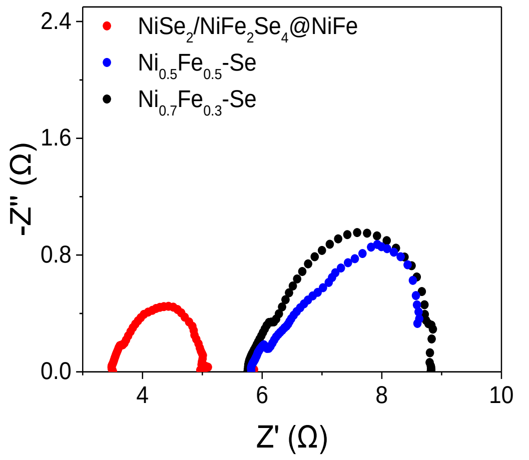

Fig. S12 Nyquist plots of $NiSe_2/NiFe_2Se_4$@NiFe, $Ni_{0.7}Fe_{0.3}$-Se, and $Ni_{0.5}Fe_{0.5}$-Se

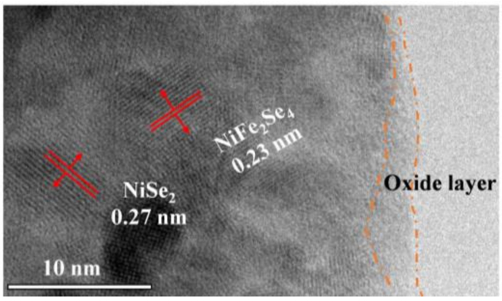

Fig. S13 HRTEM image of $NiSe_2/NiFe_2Se_4$@NiFe after OER tests

The characteristic spacing distance of 0.27 nm corresponds to the (210) plane of $NiSe_2$, while the characteristic distance of 0.23 nm is corresponded to the (211) plane of $NiFe_2Se_4$, indicating the existence of $NiSe_2$ and $NiFe_2Se_4$ in the $NiSe_2/NiFe_2Se_4$@NiFe after OER tests. Meanwhile, an amorphous oxide layer with a thickness of 1-2 nm was observed at the boundary of the $NiSe_2/NiFe_2Se_4$@NiF after OER tests, supporting the conversion of partial $NiSe_2/NiFe_2Se_4$@NiF into FeOOH and NiOOH species.

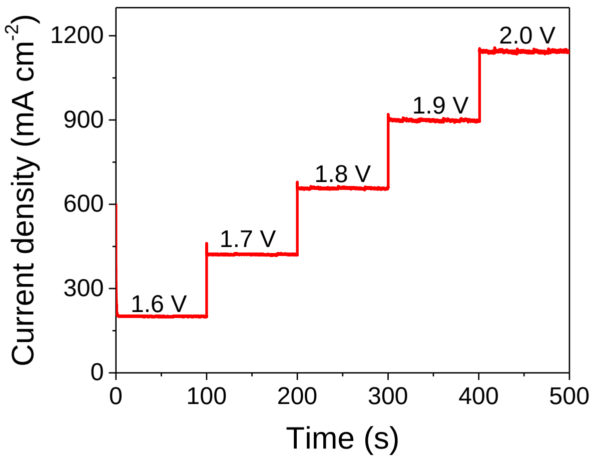

Fig. S14 Multi-potential steps curve for $NiSe_2/NiFe_2Se_4$@NiFe

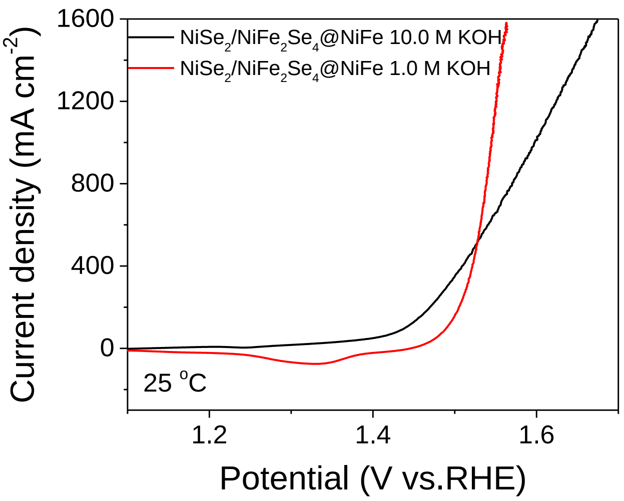

Fig. S15 Polarization curves of $NiSe_2/NiFe_2Se_4$@NiFe in 1.0 M KOH at 25 °C and 10.0 M KOH at 25 °C

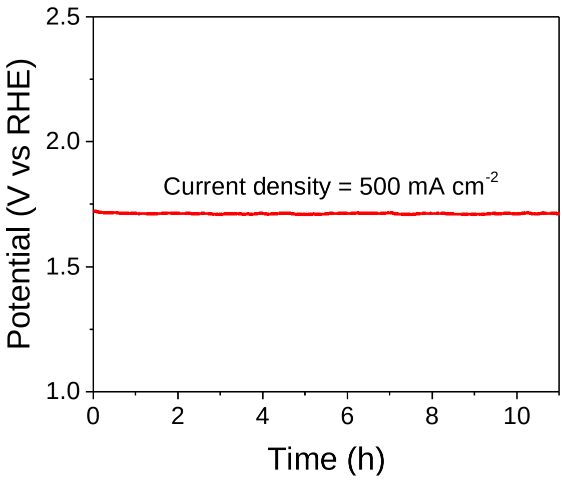

Fig. S16 Chronoamperometry curve with the $NiSe_2/NiFe_2Se_4$@NiFe as electrode at 500 mA cm^{-2} without iR compensation. Electrolyte: 1.0 M KOH

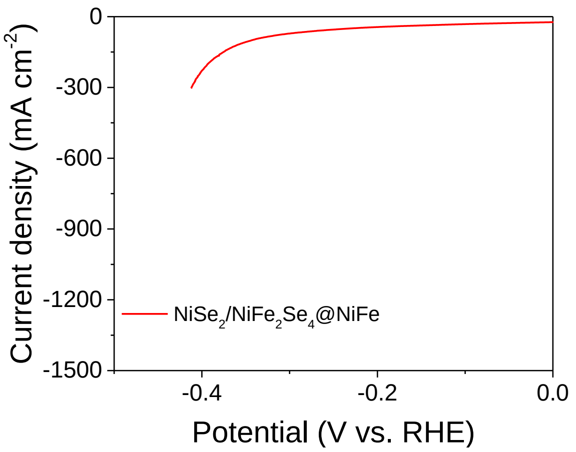

Fig. S17 Polarization curve of $NiSe_2/NiFe_2Se_4$@NiFe for HER

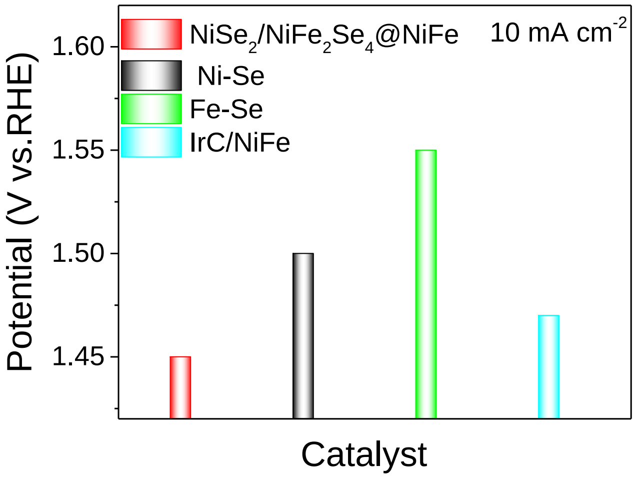

Fig. S18 The OER performances of $NiSe_2/NiFe_2Se_4$@NiFe, NF-Se, IF-Se, and Ir/C/NiFe samples to achieve current density of 10 mA cm^{-2} in 1.0 M KOH at 25 °C

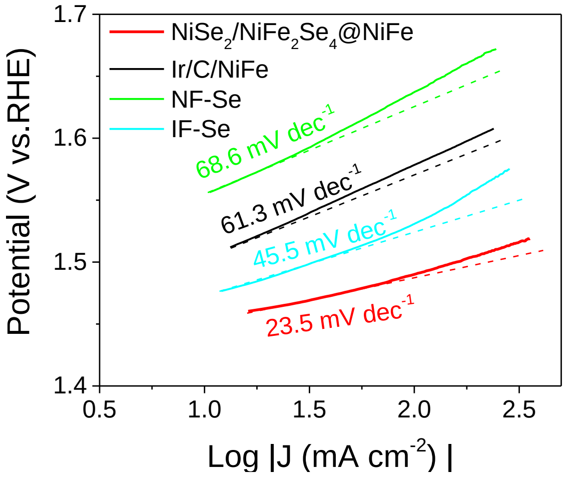

Fig. S19 Tafel plots of $NiSe_2/NiFe_2Se_4$@NiFe, NF-Se, IF-Se, and Ir/C/NiFe samples to achieve current density of 10 mA cm^{-2} in 1.0 M KOH

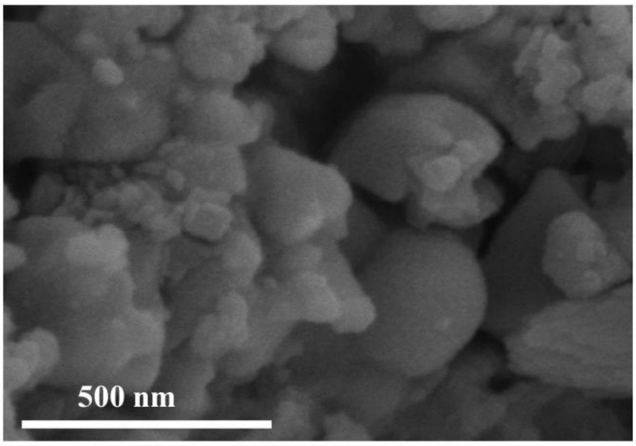

Fig. S20 FESEM image of $NiSe_2/NiFe_2Se_4$@NiFe after OER test

Table S1 OER activities of representative benchmark electrocatalysts in 1.0 M KOH in terms of the potential to achieve 100, 500, and 1,000 mA cm^{-2}

Catalyst	Electrolyte	Substrate	Tafel slope (mV dec^{-1})	Potential vs. RHE (V)		
				100 mA cm^{-2}	500 mA cm^{-2}	1,000 mA cm^{-2}
**$NiSe_2/NiFe_2Se_4$@NiFe (this work)**	**1.0 M KOH**	**NF**	**52.7**	**1.49**	**1.53**	**1.54**
$NiCoSe_2$ [S6]	1.0 M KOH	NF	97	1.55	-	-
Ni_3Se_2 [S7]	1.0 M KOH	NF	40.2	1.55	-	-
G/$NiSe_2$ [S8]	1.0 M KOH	NF	95	1.60	-	-
$CoNiSe_2$ [S9]	1.0 M KOH	NF	79	1.54	-	-
$Co_{0.13}Ni_{0.87}Se_2$ [S10]	1.0 M KOH	TI	94	1.55	-	-
$NiCo_2S_4$ [S11]	1.0 M KOH	NF	91	1.62	-	-
$NiCo_2S_4$ NCAs [S12]	1.0 M KOH	NF	68	1.58	-	-
Co_9S_8-Ni_3S_2 NAs [S13]	1.0 M KOH	NF	79.3	1.57	-	-
N-Ni_3S_2 [S14]	1.0 M KOH	NF	70	1.57	-	-
Zn-Ni_3S_2 [S15]	1.0 M KOH	NF	87	1.52	-	-
$CoSeMoS_2/Ni_3S_2$ [S16]	1.0 M KOH	NF	46.1	1.53	1.58	-
$Fe_{2.1\%}$-Ni_3S_2 [S17]	1.0 M KOH	NF	33.2	1.50	1.52	-
MoS_2-Ni_3S_2 HNRs [S18]	1.0 M KOH	NF	57	1.56	1.65	-
CDs/$NiCo_2S_4/Ni_3S_2$ [S19]	1.0 M KOH	NF	99	1.5	1.65	-
NiS [S21]	1.0 M KOH	NF	71	1.59	1.69	-
S-NiO@Ti_3C_2 [S21]	1.0 M KOH	NF	46.8	1.73	-	-

Supplementary References

[S1]S. Niu, W.J. Jiang, Z. Wei, T. Tang, J. Ma, J.S. Hu, L.J. Wan, Se-Doping activates FeOOH for cost-effective and efficient electrochemical water oxidation. J. Am. Chem. Soc. **141**(17), 7005-7013 (2019). https://doi.org/10.1021/jacs.9b01214

[S2]S. Niu, W.J. Jiang, T. Tang, L.P. Yuan, H. Luo, J.S. Hu, Autogenous growth of hierarchical NiFe(OH)x/FeS nanosheet-on-microsheet arrays for synergistically enhanced high-output water oxidation. Adv. Funct. Mater. 1902180 (2019). https://doi.org/10.1002/adfm.201902180

[S3]L. Yu, Q. Zhu, S. Song, B. McElhenny, D. Wang et al., Non-noble metal-nitride based electrocatalysts for high-performance alkaline seawater electrolysis. Nat. Commun. **10**, 5106 (2019). https://doi.org/s41467-019-13092-7

[S4]X. Cheng, C. Lei, J. Yang, B. Yang, Z. Li et al., Efficient electrocatalytic oxygen evolution at extremely high current density over 3D ultrasmall zero-valent iron-coupled nickel sulfide nanosheets. ChemElectroChem **5**, 3866-3872 (2018). https://doi.org/10.1002/celc.201801104

[S5] H. Zhou, Y. Wang, R. He, F. Yu, J. Sun, F. Wang, Y. Lan, Z. Ren, S. Chen, One-step synthesis of self-supported porous $NiSe_2$/Ni hybrid foam: An efficient 3D electrode for hydrogen evolution reaction. Nano Energy **20**, 29-36 (2016). https://doi.org/10.1016/j.nanoen.2015.12.008

[S6] K. Akbar, J.H. Jeon, M. Kim, J. Jeong, Y. Yi, S.-H. Chun, Bifunctional electrodeposited 3D $NiCoSe_2$/Nickle foam electrocatalysts for its applications in enhanced oxygen evolution reaction and for hydrazine oxidation. ACS Sustainable Chem. Eng. **6**, 7735-7742 (2018). https://doi.org/10.1021/acssuschemeng.8b00644

[S7] A. Sivanantham, S. Shanmugam, Nickel selenide supported on nickel foam as an efficient and durable non-precious electrocatalyst for the alkaline water electrolysis. Appl. Catal. B **203**, 485-493(2017). https://doi.org/10.1016/j.apcatb.2016.10.050

[S8] J. Yu, Q. Li, C.-Y. Xu, N. Chen, Y. Li, H. Liu, L. Zhen, V.P. Dravid, J. Wu, $NiSe_2$ pyramids deposited on N-doped graphene encapsulated Ni foam for high-performance water oxidation. J. Mater. Chem. A **5**, 3981-3986 (2017). https://doi.org/10.1039/C6TA10303K

[S9] T. Chen, Y. Tan, Hierarchical $CoNiSe_2$ nano-architecture as a high-performance electrocatalyst for water splitting. Nano Res. **11**, 1331-1344 (2018). https://doi.org/10.1007/s12274-017-1748-3

[S10] T. Liu, A.M. Asiri, X. Sun, Electrodeposited Co-doped $NiSe_2$ nanoparticles film: a good electrocatalyst for efficient water splitting. Nanoscale **8**, 3911-3915 (2016). https://doi.org/10.1039/C5NR07170D

[S11] X. Yin, G. Sun, L. Wang, L. Bai, L. Su, Y. Wang, Q. Du, G. Shao, 3D hierarchical network $NiCo_2S_4$ nanoflakes grown on Ni foam as efficient bifunctional electrocatalysts for both hydrogen and oxygen evolution reaction in alkaline solution. Int. J. Hydrogen Energy **42**, 25267-25276 (2017). https://doi.org/10.1016/j.ijhydene.2017.08.129

[S12] Y. Gong, J. Wang, Y. Lin, Z. Yang, H. Pan, Z. Xu, Synthesis of 1D to 3D nanostructured $NiCo_2S_4$ on nickel foam and their application in oxygen evolution reaction. Appl. Surf. Sci. **476**, 600-607 (2019). https://doi.org/10.1016/j.apsusc.2019.01.100

[S13] Y. Zhou, S. Xi, X. Yang, H. Wu, In situ hydrothermal growth of metallic Co_9S_8-Ni_3S_2 nanoarrays on nickel foam as bifunctional electrocatalysts for hydrogen and oxygen evolution reactions. J. Solid State Chem. **270**, 398-406 (2019). https://doi.org/10.1016/j.jssc.2018.12.004

[S14] P. Chen, T. Zhou, M. Zhang, Y. Tong, C. Zhong et al., 3D nitrogen-anion-decorated nickel sulfides for highly efficient overall water splitting. Adv. Mater. **29**, 1701584 (2017). https://doi.org/10.1002/adma.201701584

[S15] Q. Liu, L. Xie, Z. Liu, G. Du, A.M. Asiri, X. Sun, A Zn-doped Ni_3S_2 nanosheet array as a high-performance electrochemical water oxidation catalyst in alkaline solution. Chem. Commun. **53**, 12446-12449 (2017). https://doi.org/10.1039/C7CC06668F

[S16] W. Lu, Y. Song, M. Dou, J. Ji, F. Wang, Self-supported Ni_3S_2@MoS_2 core/shell nanorod arrays via decoration with CoS as a highly active and efficient electrocatalyst for hydrogen evolution and oxygen evolution reactions. Int. J. Hydrogen Energy **43**, 8794-8804 (2018). https://doi.org/10.1016/j.ijhydene.2018.03.110

[S17] L. Wang, Y. Li, Q. Sun, Q. Qiang, Y. Shen, Y. Ma, Z. Wang, C. Zhao, Ultralow FeIII Ion doping triggered generation of Ni_3S_2 ultrathin nanosheet for enhanced oxygen evolution reaction. ChemCatChem **11**, 2011-2016 (2019). https://doi.org/10.1002/cctc.201801959

[S18] Y. Yang, K. Zhang, H. Lin, X. Li, H.C. Chan, L. Yang, Q. Gao, MoS_2–Ni_3S_2 heteronanorods as efficient and stable bifunctional electrocatalysts for overall water splitting. ACS Catal. **7**, 2357-2366 (2017). https://doi.org/10.1021/acscatal.6b03192

[S19] X. Zhao, H. Liu, Y. Rao, X. Li, J. Wang, G. Xia, M. Wu, Carbon dots decorated hierarchical $NiCo_2S_4$/Ni_3S_2 composite for efficient water splitting. ACS Sustainable Chem. Eng. **7**, 2610-2618 (2019). https://doi.org/10.1021/acssuschemeng.8b05611

[S20] J.T. Ren, Z.Y. Yuan, Hierarchical nickel sulfide nanosheets directly grown on Ni foam: a stable and efficient electrocatalyst for water reduction and oxidation in alkaline medium. ACS Sustainable Chem. Eng. **5**, 7203-7210 (2017). https://doi.org/10.1021/acssuschemeng.7b01419

[S21] K.L. Yan, X. Shang, Z. Li, B. Dong, J.Q. Chi et al., Facile synthesis of binary NiCoS nanorods supported on nickel foam as efficient electrocatalysts for oxygen evolution reaction. Int. J. Hydrogen Energy **42**, 17129-17135 (2017). https://doi.org/10.1016/j.ijhydene.2017.05.235
